# Complete plastome sequence of *Balakata baccata* (Roxb.) Esser (Euphorbiaceae)

**DOI:** 10.1080/23802359.2021.1910083

**Published:** 2021-04-24

**Authors:** Lin-Yuan Guo, Xiao-Feng Zhang, Zhi-Xin Zhu, Hua-Feng Wang

**Affiliations:** Hainan Key Laboratory for Sustainable Utilization of Tropical Bioresources, College of Tropical Crops, Hainan University, Haikou, China

**Keywords:** *Balakata baccata*, Euphorbiaceae, plastome, genome structure

## Abstract

*Balakata baccata* belongs to the family Euphorbiaceae and is distributed in Yunnan province, China, and other southeast Asian countries, e.g., Bangladesh, Cambodia, India, Indonesia, etc. Here, we report and characterize the complete plastome of *B. baccata*. The complete plastome is 163,988 bp in length and contains a typical quadripartite structure and gene content found in angiosperms, including two inverted repeat (IR) regions of 27,274 bp, a large single-copy (LSC) region of 90,946 bp and a small single-copy (SSC) region of 18,494 bp. The plastome contains 129 genes, consisting of 84 protein-coding genes, 37 tRNA genes, and eight rRNA genes. The overall G/C content in the plastome of *B. baccata* is 35.6%. Phylogenetic results show that *B. baccata* is the earliest diverging lineage of Euphorbioideae*. Euphorbia helioscopia + E. esula, E. tirucalli + E. milii* and *B. baccata* have a closer phylogenetic relationship than other taxa within Euphorbiaceae. The complete plastome sequence of *B. baccata* will provide a useful resource for the conservation genetics of this species as well as for phylogenetic studies in Euphorbiaceae.

## Introduction

*Balakata baccata* (Roxb.) Esser (Euphorbiaceae) is a large tree species, growing up to 30 meters in height (Li et al. 2008). The species is distributed in the southern region of Yunnan province (i.e., from Pu’er to Xishuangbanna perfecture), China, and is also found in other southeast Asian countries, such as India, Myanmar, Laos, Vietnam, Cambodia, Malaysia and Indonesia (Li et al. 2008). Habitats include open forests approximately 650 meters above sea level. The complete plastome characterization and systematic position of *B. baccata* has not been reported previously. Therefore, we report and describe the complete plastome of *B. baccata* (GenBank accession number: MW266130, this study) in order to facilitate the collection, conservation and the phylogenetic studies of Euphorbiaceae and close relatives.

In this study, *B. baccata* was sampled from Ruili county in Yunnan province of China (97.77°E, 24.02°N). The voucher sample (voucher code, RL0819) and associated DNA was deposited in the Herbarium of China National GeneBank (code of herbarium: HCNGB), Hainan University, Haikou, China.

We extracted the total genomic DNA from dried leaf tissue with the cetyltrimethyl ammonium bromide (CTAB) protocol of Doyle and Doyle (1987). We analyzed genomic DNA from the sample for quantity and quality with an Agilent BioAnalyzer 2100 (UCDAVIS Genome Center, Davis, California, USA). We sheared about 0.8 μg DNA to prepare paired-end libraries with 200–400 bp insert size. We used BGISEQ-500 platform at BGI Shenzhen (China) to sequence samples, yielding about eight Gb high quality per sample with 100 bp paired-end reads. We trimmed raw reads with SOAPfilter_v2.2 (BGI-Shenzhen, China) with the following standards: at first, reads with > 10% *N*’s; second, reads with > 40% low quality bases (quality score < 10); at last, reads contaminated by adaptor sequence and produced by PCR duplication. Approximately six Gb clean data were assembled using the plastome of *Ricinus communis* (JF937588.1) (Rivarola et al. 2011) as reference with MITO bim v1.8 (Le-Petit-Quevilly, France) (Hahn et al. 2013). The assembled plastome was annotated using Geneious R11.0.4 (Biomatters Ltd., Auckland, New Zealand) with the plastome of *Euphorbia esula* (NC033910) serving as a reference. The annotation was corrected with DOGMA (Wyman et al. 2004).

The plastome of *B. baccata* is found to possess a total length 163,988 bp with the typical quadripartite structure of angiosperms, containing two Inverted Repeats (IRs) of 27,274 bp, a Large Single-Copy (LSC) region of 90,946 bp and a Small Single-Copy (SSC) region of 18,494 bp. The plastome contains 129 genes, including 84 protein-coding genes (five of which are duplicated in the IR), 37 tRNA genes (seven of which are duplicated in the IR) and eight rRNA genes (four of which are duplicated in the IR). The overall G/C content in the plastome of *B. baccata* is 35.6%, which the different regions of the LSC, SSC and IR having corresponding values of 33.1%, 29.8% and 41.8%, respectively.

**Figure 1.**
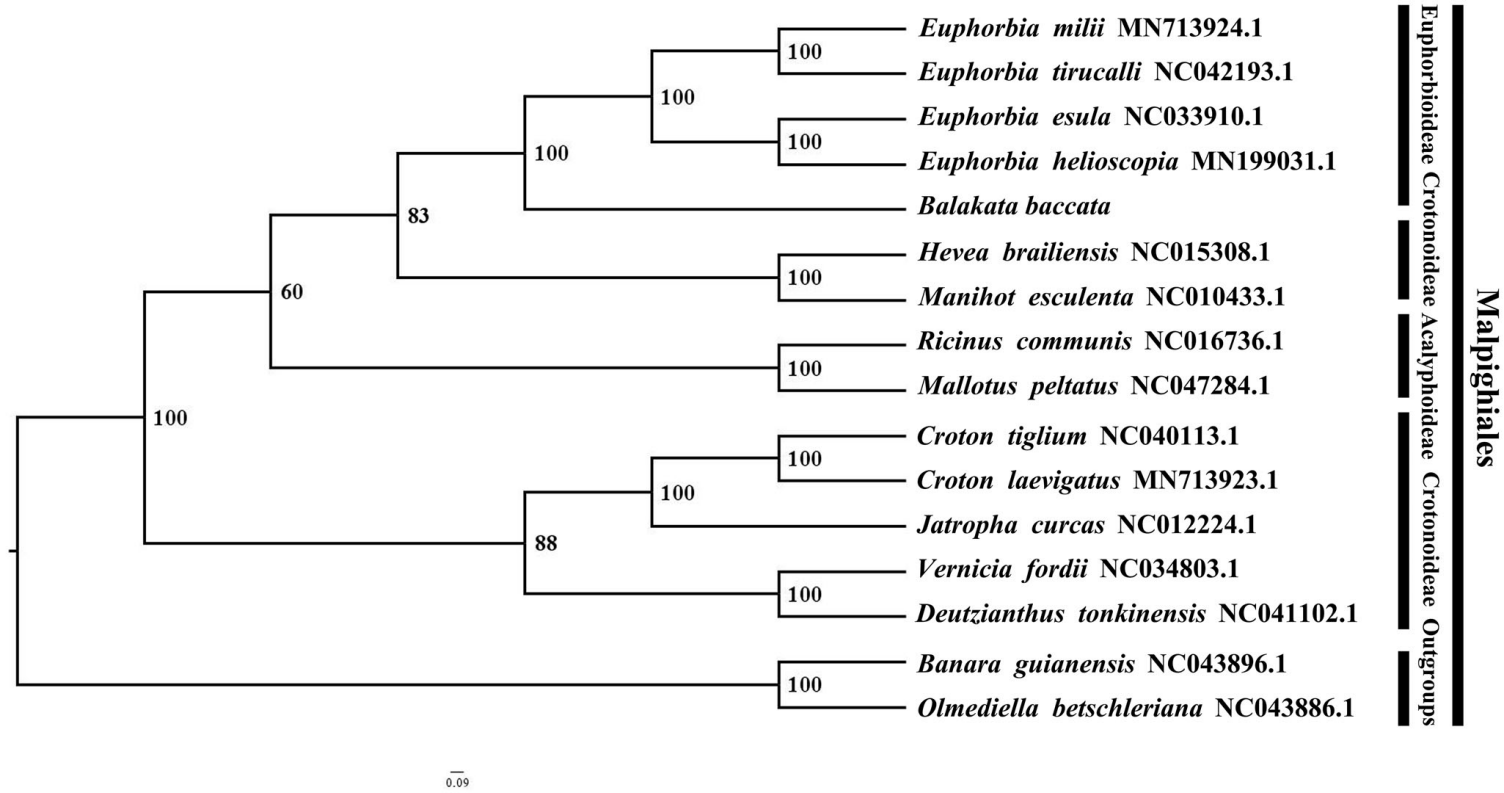
The ML phylogeny recovered from 16 complete plastome sequences by RAxML. Accession numbers: *Balakata baccata* (GenBank accession number, MW266130, this study), *Euphorbia helioscopia* MN199031.1, *Euphorbia esula* NC033910.1, *Euphorbia tirucalli* NC042193.1, *Euphorbia milii* MN73924.1, *Hevea brailiensis* NC015308.1, *Manihot esculenta* NC010433.1, *Ricinus communis* NC016736.1, *Mallotus peltatus*, NC047284.1, *Croton laevigatus* MN713923.1, *Corton tiglium* NC040113.1, *Jatropha curcas* NC012224.1, *Vernicia fordii* NC034803.1, *Deutzianthus tonkinensis* NC043896.1, *Banara guianensis* NC043896.1, *Olmediella betschleriana* NC043886.1.

We used RAxMLversion 8.4 (Stamatakis 2006) with 1,000 bootstraps using the GTRGAMMAI substitution model to reconstruct a maximum likelihood (ML) phylogeny of fourteen published complete plastomes of Euphorbiaceae, plus *Banara guianensis* (NC_043896.1) and *Olmediella betschleriana* (NC043886.1) as outgroups. By reconstructing phylogenetic relationships, we find that *Balakata baccata* is the earliest diverging taxon of Euphorbioideae*. Euphorbia helioscopia + Euphorbia esula, Euphorbia tirucalli + Euphorbia milii* and *Balakata baccata* have a closer phylogenetic relationship than other taxa within Euphorbiaceae (Figure 1). Most nodes (all but one) in the ML tree were highly supported. We increased the samples of Euphorbiaceae to increase the phylogenetic resolution and understanding of Euphorbiaceae. Furthermore, *Balakata baccata* is a plant with medicinal, ornamental and urban greening potential. The availability of a sequenced plastome will benefit its development of medicinal, ornamental and urban greening value.

## Data Availability

The genome sequence data supporting the results of this study are publicly available on GenBank of NCBI (https://www.ncbi.nlm.nih.gov/) with registration number MW266130. The associated BioProject, SRA, and Bio-Sample numbers are PRJNA438407, SRS3261059 and SAMN08770980 respectively.
